# Systems biology approaches to investigating the roles of extracellular vesicles in human diseases

**DOI:** 10.1038/s12276-019-0226-2

**Published:** 2019-03-15

**Authors:** András Gézsi, Árpád Kovács, Tamás Visnovitz, Edit I. Buzás

**Affiliations:** 1 Department of Genetics, Cell- and Immunobiology, Semmelweis University, Budapest, Hungary; 2 MTA-SE Immune-Proteogenomics Extracellular Vesicle Research Group, Budapest, Hungary; 3 Department of Measurement and Information Systems, Budapest University of Technology and Economics, Budapest, Hungary

**Keywords:** Proteomics

## Abstract

Extracellular vesicles (EVs) are membrane-enclosed structures secreted by cells. In the past decade, EVs have attracted substantial attention as carriers of complex intercellular information. They have been implicated in a wide variety of biological processes in health and disease. They are also considered to hold promise for future diagnostics and therapy. EVs are characterized by a previously underappreciated heterogeneity. The heterogeneity and molecular complexity of EVs necessitates high-throughput analytical platforms for detailed analysis. Recently, mass spectrometry, next-generation sequencing and bioinformatics tools have enabled detailed proteomic, transcriptomic, glycomic, lipidomic, metabolomic, and genomic analyses of EVs. Here, we provide an overview of systems biology experiments performed in the field of EVs. Furthermore, we provide examples of how in silico systems biology approaches can be used to identify correlations between genes involved in EV biogenesis and human diseases. Using a knowledge fusion system, we investigated whether certain groups of proteins implicated in the biogenesis/release of EVs were associated with diseases and phenotypes. Furthermore, we investigated whether these proteins were enriched in publicly available transcriptomic datasets using gene set enrichment analysis methods. We found associations between key EV biogenesis proteins and numerous diseases, which further emphasizes the key role of EVs in human health and disease.

## Diagnostics: A closer look at extracellular vesicles

Cells routinely release tiny membrane vesicles whose contents can potentially be used as indicators for various diseases. Long viewed as a system for garbage removal, researchers now recognize that these extracellular vesicles transport proteins, nucleic acids and metabolites that play an important role in cell signaling and other functions. Edit Buzás and colleagues at Semmelweis University in Hungary have reviewed the various databases that researchers have used to classify and characterize the various molecules contained within these vesicles. A number of studies indicate that the contents of vesicles might prove informative in diagnostic assays for various diseases. The authors also employed a strategy for integrating multiple extracellular vesicle datasets in order to identify genes, proteins and cell signaling pathways that represent possible connections between these vesicles and different medical conditions.

## Introduction

The rapidly emerging field of extracellular vesicles (EVs) has led to paradigm shifts in many different areas of biology and biomedicine. The release of EVs, originally thought to only act to remove harmful substances from cells, has been shown to have many more functional consequences and a wide range of implications for biomedicine. To understand the structure and function of EVs, the initial biochemical targeted approaches rapidly progressed to bias-free large-scale analyses using systems biology and bioinformatics. In 2009, the first manually curated database of EV proteins, RNA and lipids, ExoCarta^1^ (http://www.exocarta.org/), was launched. It was followed by two additional databases including Vesiclepedia^2,3^ (http://www.microvesicles.org/) and EVpedia^4,5^ (http://student4.postech.ac.kr/evpedia2_xe/xe/). These are repositories of RNA, protein, lipid, and metabolite datasets. Given that preanalytical parameters may play important roles in the quality of EV preparations, database entries should be interpreted with caution, and special attention has to be paid to preanalytical conditions. Recently, gene ontology has been extended to the context of EV communication, owing to increased recognition of the importance of the EV field^6^. Furthermore, bioinformatic tools that can be used to analyze EV datasets have become available^7,8^. Future directions may include the following: (i) systems biology analyses after more standardized EV preanalytics, (ii) multiomics analyses of EV samples (combinations of different -omic groups used for the analysis), and (iii) the determination of disease-specific EV molecular patterns/networks composed of different molecule types. Additionally, systems biology approaches may be extended to novel fields such as image-based systems biology.

Advancements in the analysis of complex biological systems such as EVs will help to reveal the biological significance of these recently discovered structures and exploit their diagnostic and/or therapeutic potential.

## EV proteomics

To date, the best characterized EV cargo is EV-associated protein molecules. Proteomics analysis of EVs has been made available on MS-based technological platforms. Proteomic analyses of EVs have been reviewed extensively elsewhere^9,10^ and are not the focus of the present article. Of note, thousands of proteins have been identified in various EV subtypes, and disease-specific proteome alterations have also been identified^11–14^. The potential for EV proteins to be used as monitoring tools for disease progression has also been successfully studied^15^. In addition, unconventional membrane protein orientation has been described in EVs^16^. The topology of various EV-associated proteins remains a very important hot topic because it influences target cell recognition by different EV subtypes and the signal transduction pathways induced by EVs.

## EV transcriptomics

A plethora of studies confirmed the feasibility of using high-throughput transcriptomic methods for EVs (such as microarrays and next-generation sequencing; see Table 1)^17–19^, and these approaches have been used successfully to characterize the healthy circulating^20,21^, urine^20,22^, cerebrospinal fluid^23^, or saliva^24,25^ EV RNA cargo. The first study exploring the physiological miRNA pattern of circulating EVs was published in 2008^26^. In the following years, the heterogeneity of circulating EV transcriptional landscapes was analyzed and revealed the presence of many different RNA types, including tRNA, miRNA, Y-RNA, mRNA, SRP-RNA, rRNA, lncRNA, piRNA, snRNA, snoRNA, and scaRNA^17,20,21^. In vitro studies further suggested that various types of RNA molecules identified in EVs were specifically shuttled into EV subsets^27^. A reference dataset for miRNA profiling in whole blood, peripheral blood cells, serum, and EVs was also published^28^. EV transcriptomics is particularly useful in the study of complex diseases because it assists in the identification of novel biomarkers (Table 1). The biomarker potential of EVs has been highlighted by high-throughput studies; however, the analysis of a single subtype of EVs^29^ instead of bulk EV ‘omics’ analyses may yield more targeted results and suggest novel therapeutic strategies.

**Table 1:** Transcriptomics of pathological condition-derived EVs

Disease/pathological condition	Biological fluid	EV transcriptomics	Applied methods	Publication
Mycobacterium tuberculosis Infection	Serum	mRNA, miRNA, snRNA, snoRNa, ncRNA,	RNA-Seq	^69^
Hand, foot, and mouth disease	Serum	miRNA	Human miRNA microarray	^70^
Type 1 diabetes mellitus	Urine	miRNA	RNA-Seq	^71^
Type 2 diabetes mellitus	Plasma	miRNA	Focus miRNA PCR panel	^72^
Heart failure after acute myocardial infarction	Serum	miRNA	miRNA microarray	^73^
Prostate cancer	Urine	mRNA	Whole-genome gene expression direct hybridization	^74^
Benign prostate hyperplasia	Urine	mRNA	Whole-genome gene expression direct hybridization	^74^
Breast cancer	Serum	miRNA	Small RNA sequencing	^75^
Breast invasive lobular carcinoma	Plasma	mRNA	RNA-Seq	^76^
Colorectal carcinoma	Plasma and serum	miRNA	Human miRNome panels I and II (Ver.3)	^77^
Ovarian cancer	Plasma	RNA	Microarray analysis	^78^
Serous papillary adenocarcinoma of the ovary	Serum	miRNA	Microarray analysis	^79^
Ovarian carcinoma	Peritoneal or pleural effusion	miRNA	TaqMan microRNA array A	^80^
Lung adenocarcinoma	Plasma	miRNA	miRCURY-human-panel-I + II-V1.M	^81^
Lung adenocarcinoma	Plasma	miRNA	miRNA microarray	^82^
Melanoma	Vitreous humor and serum	miRNA	TaqMan low density array	^83^
Pancreaticobiliary cancers	Pleural fluid and plasma	mRNA	Transcriptome sequencing	^53^
Osteosarcoma	Plasma	mRNA, rRNA, tRNA, ncRNA	RNA-Seq	^84^
Glioblastoma multiforme	Serum	mRNA	Agilent 4 × 44 K human microarrays	^85^
Young-Onset Alzheimer’s Disease	Cerebrospinal fluid	miRNA	Human miRNome panels I + II (V4.M)	^86^
Multiple sclerosis	Plasma	miRNA	3D-Gene Human miRNA oligo chip	^87^
Relapsing-remitting multiple sclerosis	Serum	tRNA, tRNA-like, rfam, miRNA, lincRNA, scaRNA, rRNA, piRNA,	XRNA RNA-seq	^88^
Cardiac allograft rejection	Serum	miRNA	Focus human microRNA PCR	^89^
Chronic lung allograft dysfunction	Bronchoalveolar lavage fluid	Exosomal shuttle RNA	RNA-seq	^90^
Oligoasthenozoospermia	Seminal plasma	miRNA	60 K microarray	^91^
Salt sensitivity of blood pressure	Urine	miRNA	miRNA microarray	^92^
Post vasectomy	Seminal plasma	miRNA	miRNA microarray	^93^
Post gastric bypass surgery	plasma and serum	miRNA	miRNA microarray	^29^

## EV metabolomics

Metabolomics involves the simultaneous detection and analysis of a large number of small molecules (<2000 Da) from biological samples^30^. The relatively low sensitivity of NMR to detect metabolites in EV samples (which are usually available in low amounts) does not allow detailed analysis of the EV metabolome. However, with advances in the available methodological platforms (e.g., Ultra-Performance Liquid Chromatography-Mass Spectrometry, UPLC MS), numerous studies have performed detailed analyses of the EV metabolome^31–34^. Interestingly, EVs have been shown to function as independent metabolic units^35^ and to modify the metabolome of their body fluid environment^36,37^ or to induce metabolic changes in recipient cells^38^.

## EV lipidomics

EV lipidomics (see Table 2) is a relatively new field mainly because the amount of an EV sample is usually very limited, and novel techniques with increased sensitivity have only recently become available to EV researchers. In the twentieth century and in the first decade of the twenty-first century, thin layer chromatography (TLC) was widely used, and it was essentially the only technique available that enabled the study of the lipid composition of EV membranes. TLC is an easy and straightforward method and does not require expensive equipment. However, the data collected in TLC experiments are very limited. Only a few lipid forms (main classes) can be separated with the help of external lipid standards. Since 2004, the application of different liquid chromatography technologies have been reported. The sensitivity and reproducibility of these experiments were significantly improved compared to those of the TLC methods, but the number of detectable lipid species was still very limited. Revolutionary development began in the early 2010s with MS-based methods, when real EV lipidomics began. The different MS-based techniques made it possible to determine the different acyl chains of membrane lipids (not just the major lipid types based on the head groups). The number of complex lipidomic studies started to increase significantly in 2016, and an exponential growth of the field is expected to come in the next few years.

**Table 2:** Lipidomic analyses of EVs

Technique for lipidomic analysis	Year of publication	EV types in the study
Thin layer chromatography (TLC)	1987^94^; 1989^95^; 1995^96^; 2002^97^; 2004^98^; 2009^99^; 2010^100^; 2015^101,102^; 2017^103^	sEV^94–96,98–103^, mEV^96,98^
Liquid chromatography (HPLC, GLC, LC-CAD)	2004;^98,104^ 2010;^100^ 2011;^105^ 2013;^106^ 2015;^102^ 2017^107^	sEV^98,100,102,104–106^, mixed EV^107^
MS-based techniques (ESI MS/MS; GC MS; LC MS/MS)	2010^100^, 2012^108^; 2013^106,109–111^; 2015^112,113^; 2016^114–116^; 2017^117–119^; 2018^120–123^	sEV^100,106,108–111,113,114,116,117,119–124^, mEV^117,118^

## EV glycomics

Glycomics in general show a relative backlog compared to other omic fields, such as genomics or proteomics (see Table 3). This is possibly due to the complexity of carbohydrate structures and the lack of sensitive and simple high-throughput methods for glycan analysis that caused a significant delay in the development of glycomics. For glycosylation analyses of EVs, lectin-based microarrays, and high resolution MS analyses have been used, and these approaches provide evidence of EV-specific glycosylation patterns.

**Table 3:** Glycomic technologies used for EV analysis

Technique for glycomic analysis	Publications	Pros and cons
Lectin-based microarrays	^125– 129^	Unbiased glycan analysis of carbohydrates on the surfaces of intact EVs
High resolution MS	^130– 134^	Requires expensive equipment. Data analysis may be time consuming

## EV genomics

Some of the EVs carry DNA that may range in size from 100 base pairs to several kilobase pairs^39^ or even fragments up to 2 million base pairs long^40^. EV-associated DNA may be single-stranded DNA, mitochondrial DNA, or double-stranded DNA^39,41,42^. The DNA content associated with EVs (termed EV-DNA) may be transported within the lumen of EVs^39,40^; however, recent studies have shown that, depending on the biological context, EV-DNA can also be found attached to the outer surface of EVs^43–46^.

Several studies have shown that EV-DNA spans sequences across all chromosomes of genomic DNA (gDNA)^39,40,47^. Sequences of mitochondrial DNA (mtDNA) may or may not be present depending on the context and/or cell line^39^. Other studies have shown that selective sorting of specific DNA sequences may occur. For example, a study investigating different prostate cancer cell-derived EV subpopulations showed that different EVs carried different gDNA contents^48^. Another study that investigated the EVs of healthy individuals provided evidence of an uneven representation of the human genome and even detected EV-DNA of bacterial origin^46^. Nevertheless, very little is currently known about the mechanisms of DNA packaging or selective sorting of DNA into EVs.

At present, the functional significance of EV-DNA is largely unknown. A recent study has shown that surface-bound EV-DNA plays a significant role in the binding of EVs to fibronectin^45^, an extracellular matrix glycoprotein that is of vital importance in processes associated with tumor progression^49^. Generally, surface-bound molecules are responsible for the binding of EVs to target cells or to the extracellular matrix^50^. Therefore, it is likely that exofacial EV-DNA may have some physiological significance for the recipient cells. Additionally, it has been shown that oncogenes can be transferred from donor to recipient cells; however, contradictory results have been reported regarding whether cancer cell-derived EV-DNA is functional in the recipient cells. In a study, the EV-mediated spread of oncogenes was shown to promote disease progression in mice^51^. Another study showed that EVs containing oncogenic H-ras failed to produce a permanent tumorigenic conversion of primary and immortalized fibroblasts^52^.

Several studies have shown that EV-DNA reflects the parental cell gDNA both qualitatively^39,47,53–58^ and quantitatively^40,42^. Therefore, the analysis of circulating EV-DNA may have substantial diagnostic potential. Moreover, the analysis of genomic mutations may prove to be superior to the analysis of the RNA transported by EVs, as DNA is intrinsically more stable than RNA.

## Systems biology approaches show relationships between genes involved in EV biogenesis and diseases

Finally, it is possible to gain information about the role of EVs through a systems biology analysis of public transcriptomic and genomic data, as well as different types of biomedical data. Our goal was to determine the relationships between key genes involved in EV biogenesis and diseases using systems biology approaches. We investigated whether a selected group of the proteins from among those reported to play a role in the biogenesis or secretion of EVs were associated with phenotypes and were enriched in publicly available transcriptomic databases.

Based on the literature, without a claim of completeness, we have compiled lists of proteins that have been reported to play roles in the biogenesis (see Tables S1 and S3) and secretion of EVs (see Table S2). We defined five partly overlapping gene sets from among these lists, namely, genes involved in EV biogenesis and secretion, EV biogenesis, exosome biogenesis, microvesicle biogenesis, and exosome secretion. We used these sets as inputs for the different analyses. Of note, the term “exosomes” refers here to small (50–150 nm in diameter) EVs that originate from the multivesicular body, whereas the designation “microvesicles” is used for EVs shed from the plasma membrane that are usually of medium size (100–1000 nm in diameter).

The Quantitative Semantic Fusion (QSF) System^59^ is an extensible framework that incorporates distinct annotated semantic types (also called entities) and links between them by integrating different data sources from the Linked Open Data world. The QSF System then enables the users to quantitatively prioritize a freely chosen entity based on evidence propagated from any other entity or possibly multiple entities through the connecting links (see Figure S1). Currently, the system contains genes, taxa, diseases, phenotypes, disease categories (UMLS semantic types and MeSH disease classes), pathways, substances, assays, cell lines, and the targets of the compounds. Links define associations between entities. For example, genes and pathways are connected with a link that represents gene-pathway associations. To enable cross-species information fusion, we also added gene orthologue links.

The most important gene-disease associations identified in this research are from the DisGeNet^60^ database. This database integrates many other sources of information (e.g., OMIM, GWAS Catalog, OrphaNet, Mouse Genome Database, and Rat Genome Database).

**Fig. 1:**
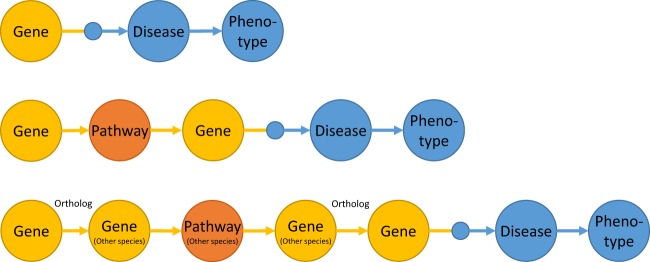
Three different models used for prioritizing the associations of key EV genes with diseases. Top: A model that prioritizes diseases and phenotypes based on gene-disease associations known in the literature. Middle: This model predicts the associated diseases and phenotypes using molecular pathway associations. Bottom: This model predicts the associated diseases and phenotypes using orthologue molecular pathway associations in other species

We constructed three different computation graphs that were used to detect known and predicted disease and phenotype associations (see Fig. 1). All three models can be used to answer the question of whether the genes involved in the biogenesis or secretion of EVs are functionally altered (for example, due to significant polymorphisms, mutations, or changes in the gene expression or the amount of protein produced), and, if so, which diseases are associated with these changes. This can also elucidate the pathomechanisms underlying the association between diseases or phenotypes and EVs.

The first model, based on gene-disease associations known in the literature (based on the data sources in the DisGeNet database), prioritizes the diseases and related phenotypes that can be linked to important genes relevant to EVs. In the second model, molecular pathway associations were used to expand the range of genes to include disease-associated genes that are in the same molecular pathways as the genes that are important for EVs. In the third model, we used the molecular pathway information from different species to predict the diseases associated with human genes the orthologues of which in other species are in the same molecular pathways as the orthologues of the human genes important for EVs.

We used the QSF System to quantitatively prioritize diseases and phenotypes that are associated with the five gene sets of genes known to be involved in the biogenesis and/or secretion of different types of EVs. First, we used a model that exploited the gene-disease associations already known in the literature. The top 20 diseases that are associated with genes that are involved either in the biogenesis or the secretion of EVs are shown in Table 4. The top 20 phenotypes are shown in Table S4. EV biogenesis genes are significantly associated with several diseases, including several tumors, such as mammary neoplasms (microvesicle biogenesis: *p* = 0.03; Exosome secretion: *p* < 0.01) and melanoma (microvesicle biogenesis: *p* = 0.02); pathologic functions, such as neoplasm invasiveness (EV biogenesis and secretion: *p* < 0.01) and neoplasm metastasis (EV biogenesis: *p* = 0.03); and cardiovascular diseases, such as myocardial reperfusion injury (microvesicle biogenesis: *p* < 0.01). The most relevant phenotypes include frontotemporal dementia (exosome biogenesis: *p* < 0.01), lack of insight (exosome biogenesis: *p* < 0.01), and autoimmune neutropenia (exosome secretion: *p* = 0.01).

**Table 4:** Diseases associated with different sets of key EV genes based on gene-disease associations known in the literature

Disease	Extracellular vesicle biogenesis and secretion		Extracellular vesicle biogenesis		Exosome biogenesis		Microvesicle biogenesis		Exosome secretion	
	Relevance score	*P*-value	Relevance score	*P*-value	Relevance score	*P*-value	Relevance score	*P*-value	Relevance score	*P*-value
Mammary neoplasms	1.00	0.00	0.94	0.05	0.36	0.35	1.00	0.03	1.00	0.00
Degenerative polyarthritis	0.72	0.00	0.55	0.00	0.34	0.04	0.41	0.03	1.00	0.00
Neoplasm invasiveness	0.71	0.00	0.82	0.00	0.33	0.18	0.83	0.00	0.60	0.00
Squamous cell carcinoma	0.66	0.00	0.37	0.02	0.04	0.27	0.52	0.00	0.84	0.00
Esophageal neoplasms	0.60	0.00	0.56	0.02	0.01	0.22	0.83	0.00	0.60	0.00
Diabetes mellitus, experimental	0.59	0.00	0.64	0.09			0.97	0.01	0.53	0.00
Neoplasm metastasis	0.53	0.00	0.82	0.03	0.53	0.03	0.62	0.03	0.29	0.07
Liver carcinoma	0.52	0.02	0.10	0.39	0.04	0.43	0.10	0.15	0.81	0.00
Non-small cell lung carcinoma	0.49	0.00	0.29	0.12	0.00	0.91	0.45	0.03	0.61	0.00
Mouth neoplasms	0.48	0.00							0.80	0.00
Melanoma	0.47	0.02	0.68	0.02	0.39	0.10	0.55	0.02	0.30	0.05
Animal mammary neoplasms	0.47	0.00							0.79	0.00
Juvenile-onset dystonia	0.47	0.01							0.79	0.00
IGA glomerulonephritis	0.44	0.07	1.00	0.00	1.00	0.05	0.28	0.32	0.20	0.12
Mammary neoplasms, experimental	0.43	0.01	0.18	0.28			0.28	0.19	0.60	0.00
Alzheimer’s disease	0.43	0.06	0.32	0.15	0.01	0.74	0.48	0.06	0.49	0.02
Prostatic neoplasms	0.42	0.11	0.07	0.65	0.01	0.77	0.10	0.30	0.65	0.00
Stomach neoplasms	0.41	0.05	0.07	0.49	0.02	0.46	0.07	0.20	0.63	0.00
Adenocarcinoma	0.40	0.01	0.33	0.08	0.00	0.75	0.48	0.02	0.43	0.01
Myocardial reperfusion injury	0.39	0.02	0.64	0.00	0.22	0.17	0.97	0.00	0.20	0.11

For each gene list (columns), the relevance score is the normalized relevance score computed by the first model (see Fig. 1). *P*-values were computed by permutation tests. The top 20 most relevant diseases are reported based on the gene list of EV biogenesis and secretion

Pathway-mediated analysis (i.e., determining which diseases are associated with genes that participate in the same pathway as EV biogenesis genes) indicated possible associations of EVs with many common diseases (see Table S5 and Table S6), such as diabetes (microvesicle biogenesis: *p* < 0.01), Alzheimer’s disease (EV biogenesis and secretion: *p* < 0.01), and obesity (microvesicle biogenesis: *p* = 0.02). Cross-species pathway-mediated analysis indicated the possible association of EVs with several tumors (see Tables S7 and S8), such as mouth neoplasms (exosome secretion: *p* < 0.01) and tongue neoplasms (exosome secretion: *p* < 0.01) and several other diseases and conditions.

Next, we downloaded and reanalyzed five large publicly available microarray data sets from the Gene Expression Omnibus (GEO) that represent various diseases (accessions: GSE13576, GSE6919, GSE4115, GSE54514, and GSE43696). Then, we computed the enrichment of the five key EV gene sets and all KEGG pathways in the various contrasts of the differential expression analyses. The statistical analyses were performed in R statistical language^61^. We used the limma^62^ and EGSEA^63^ packages for the microarray and enrichment analysis, respectively.

The Ensemble of Gene Set Enrichment Analysis (EGSEA) utilizes and combines the analysis results of many prominent gene set enrichment algorithms to calculate the collective significance score for a given gene set in the generally long lists of genes that arise from a differential expression analysis.

We reanalyzed five publicly available gene expression experiments using contrasts defined by the authors of these experiments, and then we computed the enrichment of the five key EV gene lists using the EGSEA method based on these contrasts (i.e., gene expression signatures relevant for a specific biological process).

The key EV gene sets were statistically significantly enriched in many of the analyzed contrasts (see Table 5).

**Table 5:** Enrichment of different sets of key EV genes in various gene expression experiments

GEO accession	Study	Contrast	Extracellular vesicle biogenesis and secretion	Extracellular vesicle biogenesis	Exosome biogenesis	Microvesicle biogenesis	Exosome secretion
GSE13576	Xenografted leukemia samples with different time to leukemia phenotypes	No relapse vs. early relapse	**1.16E-11**	**1.24E-05**	**3.27E-04**	**1.14E-02**	**2.66E-06**
		No relapse vs. late relapse	**3.46E-03**	1.56E-01	2.53E-01	7.43E-01	9.51E-02
		No relapse vs. relapse	**7.85E-15**	**5.25E-07**	**2.30E-05**	**3.78E-03**	**6.53E-08**
		Early relapse vs. late relapse	**5.11E-03**	1.71E-01	3.24E-01	7.79E-01	1.37E-01
GSE6919	Normal and prostate tumor tissues	Healthy vs. tumor	**1.59E-03**	**3.45E-02**	4.29E-01	9.54E-02	**8.41E-04**
		Tumor vs. adjacent	**4.70E-03**	6.29E-01	9.88E-01	8.70E-01	**1.34E-03**
		Tumor vs. metastatic	**1.58E-09**	**9.75E-04**	**1.87E-02**	**8.04E-06**	**4.55E-11**
GSE4115	Smokers with suspected lung cancer	No cancer vs. cancer	**5.53E-25**	**3.48E-08**	**3.37E-04**	**8.37E-06**	**2.40E-21**
GSE54514	Survivors and nonsurvivors of sepsis	Healthy vs. survivor	**1.32E-16**	**1.99E-03**	**2.81E-02**	9.59E-02	**1.51E-15**
		Healthy vs. nonsurvivor	**7.00E-09**	**1.79E-05**	**1.67E-03**	**4.87E-05**	**1.26E-07**
		Healthy vs. sepsis (survivor + nonsurvivor)	**1.92E-25**	**2.11E-05**	**2.00E-03**	5.41E-01	**3.42E-23**
		Nonsurvivor vs. survivor	**3.79E-25**	**9.98E-05**	**1.28E-03**	**3.64E-09**	**3.99E-24**
GSE43696	Normal controls, mild-moderate asthmatic patients and severe asthmatic patients	Control vs. moderate asthma	9.78E-01	9.21E-01	9.94E-01	9.21E-01	1.00E + 00
		Control vs. severe asthma	5.62E-02	1.96E-01	3.43E-01	3.34E-01	5.62E-02
		Control vs. asthma (moderate + severe)	4.40E-01	6.16E-01	8.09E-01	7.70E-01	3.96E-01
		Moderate vs. severe asthma	2.54E-01	3.53E-01	4.23E-01	5.08E-01	3.74E-01

Values in the table represent Benjamini-Hochberg adjusted *p*-values for enrichment combining the results of 10 different enrichment algorithms. Statistically significant *p*-values (<0.05) are in bold

Meyer et al. investigated the engraftment properties and impact on outcomes of 50 pediatric acute lymphoblastic leukemia samples transplanted into NOD/SCID mice^64^. They found that the time to the development of leukemia (i.e., weeks from transplant to overt leukemia) was strongly associated with the risk of early relapse. We found that the differentially expressed genes between the no relapse and the early relapse groups were significantly enriched for key EV genes as well.

Yu et al. performed a comprehensive gene expression analysis on 152 human samples, including prostate cancer tissues, prostate tissues adjacent to tumor, and organ donor prostate tissues, obtained from men of various ages^65^. The differentially expressed genes between the nonmetastatic tumor samples and the metastatic tumor samples were significantly enriched for all key EV gene sets.

Spira et al. compared gene expression data from smokers with lung cancer with samples from smokers without lung cancer^66^. This allowed them to generate a diagnostic gene expression profile that could distinguish between the two classes. We found that all EV gene sets were significantly enriched in the gene expression profile comparing smokers with and without lung cancer.

Parnell et al. performed gene expression profiling of whole blood to monitor immune dysfunction in critically ill septic patients^67^. We found that all gene expression signatures comparing healthy controls with sepsis survivors, healthy controls with nonsurvivors, and nonsurvivors with survivors were significantly enriched for EV genes.

Voraphani et al. compared the gene expression profiles of airway epithelial and bronchoalveolar lavage cells of healthy controls, mild-moderate asthmatic patients, and severe refractory asthmatic patients, respectively^68^. We found no enrichment in the different gene expression signatures.

Genes that have been reported to participate in the biogenesis or secretion of EVs are significantly associated with numerous common diseases, including different types of tumors and cardiovascular diseases, which further emphasizes the key role of EVs in human health and disease.

## Supplementary information

Supplementary Information
