# Supplementary material for: Systems biology approaches to investigating the roles of extracellular vesicles in human diseases
Source: Exp Mol Med. 2019 Mar 15;51(3):33. doi: 10.1038/s12276-019-0226-2 (PMC6418293; doi:10.1038/s12276-019-0226-2)
Supplement: Supplementary file 1 — Supplementary Information [file 12276_2019_226_MOESM1_ESM.docx]

# Supplementary Information

**Figure S1.** **Quantitative Semantic Fusion (QSF) System.** (A) The QSF System incorporates distinct annotated semantic types (i.e., entities) and their quantitative pairwise relations (i.e., links) by integrating different data sources from the Linked Open Data world. Predefined entities and links from DisGeNET, Ensembl, ChEMBL and WikiPathways are shown at the top. (B) The user can freely construct so-called computation graphs using the available entities and links and can select any entity as the target of the prioritization. An example computation graph is shown in panel B. The system propagates the evidence for the selected entities through the links to the target entity and quantitatively prioritizes all elements of that entity. (C) The explanation of the results of the prioritization is represented by the system in the form of networks.

| 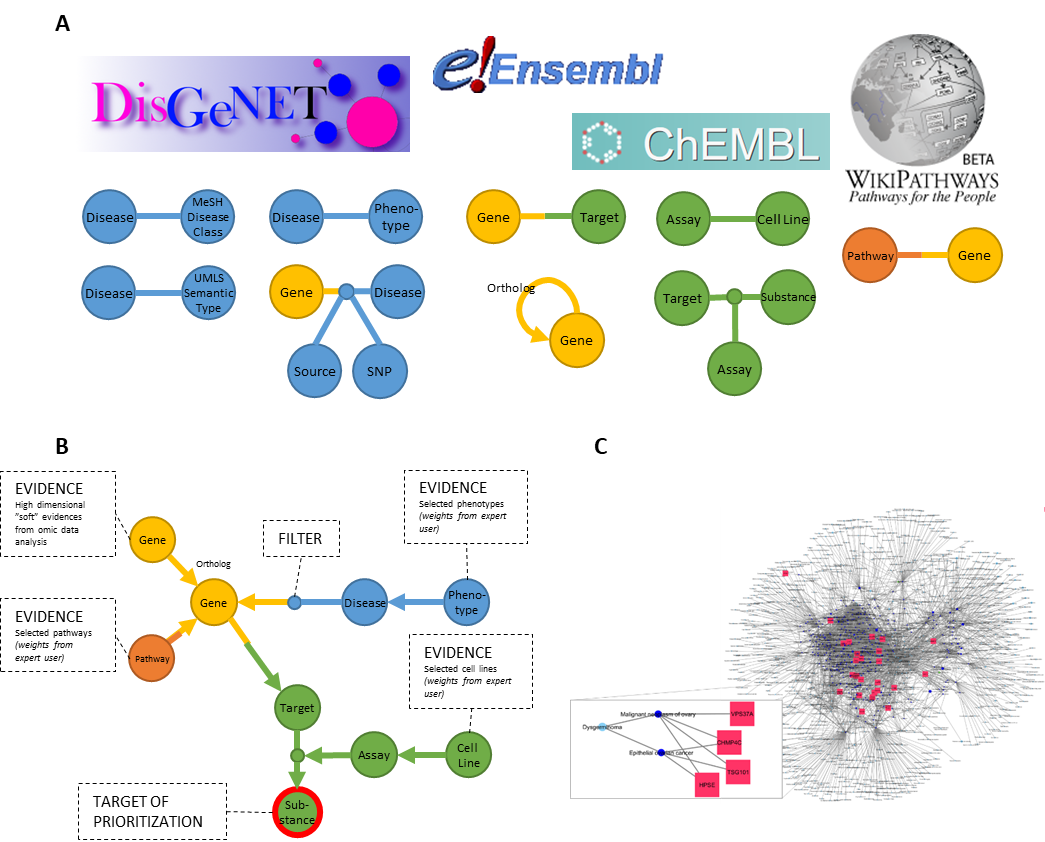 | |
| --- | --- |
|  |  |

**Table S1.** Genes that play a role in the biogenesis of multivesicular body-derived exosomes

| **Gene** | **Ensembl identifier** | **Entrez** | **Alternative name** |
| --- | --- | --- | --- |
| HGS | ENSG00000185359 | 9146 | HRS |
| STAM | ENSG00000136738 | 8027 | STAM1; STAM-1 |
| STAM2 | ENSG00000115145 | 10254 | Hbp; STAM2A; STAM2B |
| VPS28 | ENSG00000160948 | 51160 |  |
| VPS37A | ENSG00000155975 | 137492 | HCRP1; PQBP2; SPG53 |
| VPS37B | ENSG00000139722 | 79720 |  |
| VPS37C | ENSG00000167987 | 55048 |  |
| VPS37D | ENSG00000176428 | 155382 | WBSCR24 |
| MVB12A | ENSG00000141971 | 93343 | CFBP; FAM125A |
| MVB12B | ENSG00000196814 | 89853 | C9orf28; FAM125B |
| SNF8 | ENSG00000159210 | 11267 | Dot3; EAP30; VPS22 |
| VPS25 | ENSG00000131475 | 84313 | DERP9; EAP20; FAP20 |
| VPS36 | ENSG00000136100 | 51028 | EAP45; C13orf9; CGI-145 |
| CHMP1A | ENSG00000131165 | 5119 | PCH8; CHMP1; PRSM1; PCOLN3; VPS46A; VPS46-1 |
| CHMP1B | ENSG00000255112 | 57132 | Vps46B; C10orf2; C18orf2; CHMP1.5; Vps46-2; C18-ORF2; hVps46-2 |
| CHMP2A | ENSG00000130724 | 27243 | BC2; BC-2; VPS2; CHMP2; VPS2A |
| CHMP2B | ENSG00000083937 | 25978 | DMT1; ALS17; VPS2B; VPS2-2; CHMP2.5 |
| CHMP3 | ENSG00000115561 | 51652 | NEDF; VPS24; CGI-149 |
| CHMP4A | ENSG00000254505 | 29082 | SNF7; CHMP4; SHAX2; CHMP4B; SNF7-1; VPS32A; HSPC134; VPS32-1; C14orf123 |
| CHMP4B | ENSG00000101421 | 128866 | SNF7; CTPP3; Shax1; CHMP4A; SNF7-2; VPS32B; CTRCT31; Vps32-2; C20orf178; dJ553F4.4 |
| CHMP4C | ENSG00000164695 | 92421 | Shax3; SNF7-3; VPS32C |
| CHMP5 | ENSG00000086065 | 51510 | Vps60; CGI-34; PNAS-2; C9orf83; HSPC177; SNF7DC2 |
| CHMP6 | ENSG00000176108 | 79643 | VPS20 |
| PDCD6IP | ENSG00000170248 | 10015 | AIP1; ALIX; HP95; DRIP4 |
| IST1 | ENSG00000182149 | 9798 | OLC1 |
| TSG101 | ENSG00000074319 | 7251 | TSG10; VPS23 |
| VPS4A | ENSG00000132612 | 27183 | SKD1; SKD2; VPS4; SKD1A; VPS4-1 |
| HPSE | ENSG00000173083 | 10855 | HPA; HPA1; HPR1; HSE1; HPSE1 |
| ARF6 | ENSG00000165527 | 382 |  |
| PLD2 | ENSG00000129219 | 5338 | PLD1C |

**Table S2.** Genes that play a role in the secretion of multivesicular body-derived exosomes

| **Gene** | **Ensembl identifier** | **Entrez** | **Alternative name** |
| --- | --- | --- | --- |
| PDCD6IP | ENSG00000170248 | 10015 | AIP1; ALIX; HP95; DRIP4 |
| EEF1A1 | ENSG00000156508 | 1915 | CCS3; EF1A; PTI1; CCS-3; EE1A1; EEF-1; EEF1A; EF-Tu; LENG7; eEF1A-1; GRAF-1EF; HNGC:16303 |
| ENO1 | ENSG00000074800 | 2023 | NNE; PPH; MPB1; ENO1L1; HEL-S-17 |
| FLOT2 | ENSG00000132589 | 2319 | ESA; ECS1; ESA1; ECS-1; M17S1 |
| GAPDH | ENSG00000111640 | 2597 | G3PD; GAPD; HEL-S-162eP |
| ANXA2 | ENSG00000182718 | 302 | P36; ANX2; LIP2; LPC2; CAL1H; LPC2D; ANX2L4; PAP-IV; HEL-S-270 |
| HSPA8 | ENSG00000109971 | 3312 | LAP1; HSC54; HSC70; HSC71; HSP71; HSP73; LAP-1; NIP71; HEL-33; HSPA10; HEL-S-72p |
| HSP90AA1 | ENSG00000080824 | 3320 | EL52; HSPN; LAP2; HSP86; HSPC1; HSPCA; Hsp89; Hsp90; LAP-2; HSP89A; HSP90A; HSP90N; Hsp103; HSPCAL1; HSPCAL4; HEL-S-65p |
| FASLG | ENSG00000117560 | 356 | APTL; FASL; CD178; CD95L; ALPS1B; CD95-L; TNFSF6; TNLG1A; APT1LG1 |
| LAMP1 | ENSG00000185896 | 3916 | LAMPA; CD107a; LGP120 |
| ACHE | ENSG00000087085 | 43 | YT; ACEE; ARACHE; N-ACHE |
| ACTB | ENSG00000075624 | 60 | BRWS1; PS1TP5BP1 |
| SDC1 | ENSG00000115884 | 6382 | SDC; CD138; SYND1; syndecan |
| SDC2 | ENSG00000169439 | 6383 | HSPG; CD362; HSPG1; SYND2 |
| SDCBP | ENSG00000137575 | 6386 | ST1; MDA9; SYCL; MDA-9; TACIP18 |
| TFRC | ENSG00000072274 | 7037 | T9; TR; TFR; p90; CD71; TFR1; TRFR; IMD46 |
| TSG101 | ENSG00000074319 | 7251 | TSG10; VPS23 |
| CD9 | ENSG00000010278 | 928 | MIC3; MRP-1; BTCC-1; DRAP-27; TSPAN29; TSPAN-29 |
| CD63 | ENSG00000135404 | 967 | MLA1; ME491; LAMP-3; OMA81H; TSPAN30 |
| CD81 | ENSG00000110651 | 975 | S5.7; CVID6; TAPA1; TSPAN28 |
| MLKL | ENSG00000168404 | 197259 | hMLKL |
| GIPC1 | ENSG00000123159 | 10755 | NIP; GIPC; IIP-1; TIP-2; SEMCAP; C19orf3; Hs.6454; GLUT1CBP; RGS19IP1; SYNECTIN; SYNECTIIN |

**Table S3.** Genes that play a role in the biogenesis of plasma membrane-derived microvesicles

| **Gene** | **Ensembl identifier** | **Entrez** | **Alternative name** |
| --- | --- | --- | --- |
| CASP3 | ENSG00000164305 | 836 | CPP32; SCA-1; CPP32B |
| CAPN1 | ENSG00000014216 | 823 | CANP; muCL; CANP1; SPG76; CANPL1; muCANP |
| RAB22A | ENSG00000124209 | 57403 |  |
| ARRDC1 | ENSG00000197070 | 92714 |  |
| TSG101 | ENSG00000074319 | 7251 | TSG10; VPS23 |
| ARF6 | ENSG00000165527 | 382 |  |
| PLD2 | ENSG00000129219 | 5338 | PLD1C |
| TGM2 | ENSG00000198959 | 7052 | TGC; TG(C) |
| VAMP3 | ENSG00000049245 | 9341 | CEB |
| ITGB1 | ENSG00000150093 | 3688 | CD29; FNRB; MDF2; VLAB; GPIIA; MSK12; VLA-BETA |
| MMP14 | ENSG00000157227 | 4323 | MMP-14; MMP-X1; MT-MMP; MT1MMP; MTMMP1; WNCHRS; MT1-MMP; MT-MMP 1 |

**Table S4. Phenotypes associated with different sets of key EV genes based on gene-disease and disease-phenotype associations known in the literature.** For each gene list (columns), the relevance score is the normalized relevance score computed by the first model (see Figure 1). P-values are computed by permutation tests. The top 20 most relevant phenotypes are reported based on the gene list of EV biogenesis and secretion.

| Phenotype | Extracellular vesicle biogenesis and secretion | | Extracellular vesicle biogenesis | | Exosome biogenesis | | Microvesicle biogenesis | | Exosome secretion | |
| --- | --- | --- | --- | --- | --- | --- | --- | --- | --- | --- |
|  | Relevance score | P-value | Relevance score | P-value | Relevance score | P-value | Relevance score | P-value | Relevance score | P-value |
| Externally rotated hips | 1.00 | 0.00 |  |  |  |  |  |  | 1.00 | 0.00 |
| Subacute progressive viral hepatitis | 0.64 | 0.03 | 0.12 | 0.39 | 0.03 | 0.38 | 0.10 | 0.15 | 0.59 | 0.00 |
| Antinuclear antibody positivity | 0.60 | 0.00 | 0.20 | 0.18 |  |  | 0.33 | 0.07 | 0.50 | 0.00 |
| Lack of insight | 0.58 | 0.01 | 0.88 | 0.00 | 0.90 | 0.00 |  |  | 0.14 | 0.01 |
| Mild global developmental delay | 0.58 | 0.01 |  |  |  |  |  |  | 0.58 | 0.01 |
| Alveolar cell carcinoma | 0.55 | 0.01 | 0.24 | 0.13 | 0.01 | 0.56 | 0.37 | 0.04 | 0.43 | 0.01 |
| Autoimmune neutropenia | 0.54 | 0.00 |  |  |  |  |  |  | 0.54 | 0.01 |
| Antiphospholipid antibody positivity | 0.50 | 0.00 | 0.00 | 0.74 |  |  | 0.00 | 0.45 | 0.50 | 0.00 |
| Generalized dystonia | 0.50 | 0.00 |  |  |  |  |  |  | 0.50 | 0.01 |
| Frontotemporal dementia | 0.49 | 0.04 | 1.00 | 0.00 | 1.00 | 0.00 | 0.00 | 0.03 | 0.00 | 0.03 |
| Decreased resting energy expenditure | 0.48 | 0.24 | 0.36 | 0.27 | 0.37 | 0.22 | 0.00 | 0.55 | 0.30 | 0.18 |
| Chronic noninfectious lymphadenopathy | 0.46 | 0.00 |  |  |  |  |  |  | 0.46 | 0.00 |
| Increased number of peripheral CD3+ T cells | 0.46 | 0.00 |  |  |  |  |  |  | 0.46 | 0.00 |
| Increased number of CD4-/CD8- T cells expressing alpha/beta T-cell receptors | 0.46 | 0.00 |  |  |  |  |  |  | 0.46 | 0.00 |
| Increased proportion of HLA DR+ and CD57+ T cells | 0.46 | 0.00 |  |  |  |  |  |  | 0.46 | 0.00 |
| Rheumatoid factor positive | 0.46 | 0.00 |  |  |  |  |  |  | 0.46 | 0.00 |
| Increased IgG level | 0.46 | 0.00 |  |  |  |  |  |  | 0.46 | 0.00 |
| Smooth muscle antibody positivity | 0.46 | 0.00 |  |  |  |  |  |  | 0.46 | 0.00 |
| Antineutrophil antibody positivity | 0.46 | 0.00 |  |  |  |  |  |  | 0.46 | 0.00 |
| Hip osteoarthritis | 0.46 | 0.01 | 0.32 | 0.00 | 0.16 | 0.02 | 0.23 | 0.04 | 0.38 | 0.01 |
| Frontal release signs | 0.45 | 0.01 | 0.68 | 0.00 | 0.69 | 0.00 | 0.00 | 0.05 | 0.11 | 0.01 |

**Table S5. Diseases associated with different sets of key EV genes based on pathway-mediated gene-disease associations.** For each gene list (columns), the relevance score is the normalized relevance score computed by the second model (see Figure 1). P-values are computed by permutation tests. The top 20 most relevant diseases are reported based on the gene list of EV biogenesis and secretion.

| Disease | Extracellular vesicle biogenesis and secretion | | Extracellular vesicle biogenesis | | Exosome biogenesis | | Microvesicle biogenesis | | Exosome secretion | |
| --- | --- | --- | --- | --- | --- | --- | --- | --- | --- | --- |
|  | Relevance score | P-value | Relevance score | P-value | Relevance score | P-value | Relevance score | P-value | Relevance score | P-value |
| Mammary neoplasms | 1.00 | 0.00 | 1.00 | 0.00 | 0.99 | 0.61 | 1.00 | 0.00 | 1.00 | 0.04 |
| Diabetes mellitus, experimental | 0.89 | 0.00 | 0.83 | 0.00 | 0.77 | 0.73 | 0.83 | 0.00 | 0.97 | 0.03 |
| Diabetes mellitus, non-insulin-dependent | 0.83 | 0.01 | 0.76 | 0.02 | 1.00 | 0.47 | 0.76 | 0.00 | 0.91 | 0.04 |
| Prostatic neoplasms | 0.78 | 0.00 | 0.77 | 0.00 | 0.77 | 0.62 | 0.77 | 0.00 | 0.79 | 0.04 |
| Hypertensive disease | 0.73 | 0.03 | 0.72 | 0.01 | 0.68 | 0.70 | 0.72 | 0.00 | 0.75 | 0.14 |
| Reperfusion injury | 0.70 | 0.00 | 0.71 | 0.00 | 0.53 | 0.70 | 0.71 | 0.00 | 0.70 | 0.03 |
| Liver carcinoma | 0.69 | 0.00 | 0.68 | 0.00 | 0.75 | 0.59 | 0.68 | 0.00 | 0.71 | 0.04 |
| Stomach neoplasms | 0.66 | 0.00 | 0.66 | 0.00 | 0.53 | 0.73 | 0.66 | 0.00 | 0.65 | 0.04 |
| Asthma | 0.63 | 0.09 | 0.60 | 0.10 | 0.23 | 0.87 | 0.60 | 0.07 | 0.66 | 0.21 |
| Rheumatoid arthritis | 0.61 | 0.04 | 0.57 | 0.06 | 0.14 | 0.98 | 0.57 | 0.00 | 0.66 | 0.11 |
| Neoplasm metastasis | 0.57 | 0.00 | 0.61 | 0.00 | 0.66 | 0.45 | 0.61 | 0.00 | 0.53 | 0.07 |
| Myocardial infarction | 0.57 | 0.03 | 0.57 | 0.00 | 0.26 | 0.86 | 0.57 | 0.00 | 0.57 | 0.08 |
| Alzheimer's disease | 0.57 | 0.00 | 0.57 | 0.00 | 0.26 | 0.82 | 0.57 | 0.00 | 0.56 | 0.03 |
| Lung neoplasms | 0.55 | 0.00 | 0.57 | 0.00 | 0.52 | 0.60 | 0.57 | 0.00 | 0.52 | 0.08 |
| Schizophrenia | 0.53 | 0.03 | 0.51 | 0.01 | 0.30 | 0.88 | 0.51 | 0.00 | 0.55 | 0.06 |
| Obesity | 0.52 | 0.09 | 0.49 | 0.05 | 0.62 | 0.71 | 0.49 | 0.02 | 0.56 | 0.23 |
| Brain ischemia | 0.52 | 0.00 | 0.55 | 0.00 | 0.27 | 0.75 | 0.55 | 0.00 | 0.48 | 0.03 |
| Diabetes mellitus, insulin-dependent | 0.49 | 0.13 | 0.46 | 0.14 | 0.17 | 0.79 | 0.46 | 0.08 | 0.53 | 0.25 |
| Melanoma | 0.47 | 0.00 | 0.50 | 0.00 | 0.56 | 0.43 | 0.50 | 0.00 | 0.45 | 0.07 |
| Colonic neoplasms | 0.45 | 0.00 | 0.45 | 0.00 | 0.32 | 0.70 | 0.45 | 0.00 | 0.45 | 0.05 |

**Table S6. Phenotypes associated with different sets of key EV genes based on pathway-mediated gene-disease and disease-phenotype associations.** For each gene list (columns), the relevance score is the normalized relevance score computed by the second model (see Figure 1). P-values are computed by permutation tests. The top 20 most relevant phenotypes are reported based on the gene list of EV biogenesis and secretion.

| Phenotype | Extracellular vesicle biogenesis and secretion | | Extracellular vesicle biogenesis | | Exosome biogenesis | | Microvesicle biogenesis | | Exosome secretion | |
| --- | --- | --- | --- | --- | --- | --- | --- | --- | --- | --- |
|  | Relevance score | P-value | Relevance score | P-value | Relevance score | P-value | Relevance score | P-value | Relevance score | P-value |
| Long-tract signs | 0.53 | 0.03 | 0.55 | 0.03 | 0.21 | 0.87 | 0.55 | 0.08 | 0.51 | 0.08 |
| Senile plaques | 0.33 | 0.04 | 0.34 | 0.03 | 0.11 | 0.86 | 0.35 | 0.08 | 0.31 | 0.08 |
| Follicular hyperplasia | 0.20 | 0.01 | 0.17 | 0.03 | 0.05 | 0.42 | 0.15 | 0.06 | 0.23 | 0.00 |
| Defective lymphocyte apoptosis | 0.20 | 0.01 | 0.17 | 0.03 | 0.05 | 0.42 | 0.15 | 0.06 | 0.23 | 0.00 |
| Chronic noninfectious lymphadenopathy | 0.19 | 0.02 | 0.17 | 0.03 |  |  | 0.15 | 0.06 | 0.21 | 0.01 |
| Increased number of peripheral CD3+ T cells | 0.19 | 0.02 | 0.17 | 0.03 |  |  | 0.15 | 0.06 | 0.21 | 0.01 |
| Increased number of CD4-/CD8- T cells expressing alpha/beta T-cell receptors | 0.19 | 0.02 | 0.17 | 0.03 |  |  | 0.15 | 0.06 | 0.21 | 0.01 |
| Increased proportion of HLA DR+ and CD57+ T cells | 0.19 | 0.02 | 0.17 | 0.03 |  |  | 0.15 | 0.06 | 0.21 | 0.01 |
| Rheumatoid factor positive | 0.19 | 0.02 | 0.17 | 0.03 |  |  | 0.15 | 0.06 | 0.21 | 0.01 |
| Increased IgG level | 0.19 | 0.02 | 0.17 | 0.03 |  |  | 0.15 | 0.06 | 0.21 | 0.01 |
| Smooth muscle antibody positivity | 0.19 | 0.02 | 0.17 | 0.03 |  |  | 0.15 | 0.06 | 0.21 | 0.01 |
| Antineutrophil antibody positivity | 0.19 | 0.02 | 0.17 | 0.03 |  |  | 0.15 | 0.06 | 0.21 | 0.01 |
| Platelet antibody positive | 0.18 | 0.02 | 0.16 | 0.03 |  |  | 0.15 | 0.06 | 0.20 | 0.01 |
| Coombs-positive hemolytic anemia | 0.17 | 0.02 | 0.15 | 0.03 |  |  | 0.15 | 0.06 | 0.19 | 0.01 |
| Autoimmune neutropenia | 0.17 | 0.01 | 0.15 | 0.03 |  |  | 0.15 | 0.08 | 0.18 | 0.01 |
| Increased IgA level | 0.16 | 0.03 | 0.14 | 0.06 |  |  | 0.15 | 0.12 | 0.19 | 0.04 |
| Increased IgM level | 0.15 | 0.04 | 0.14 | 0.07 |  |  | 0.15 | 0.07 | 0.17 | 0.03 |
| Reduced delayed hypersensitivity | 0.15 | 0.02 | 0.14 | 0.05 |  |  | 0.15 | 0.07 | 0.17 | 0.01 |
| Hepatic amyloidosis | 0.13 | 0.04 | 0.14 | 0.03 |  |  | 0.15 | 0.03 | 0.12 | 0.06 |
| Fasciitis | 0.13 | 0.04 | 0.14 | 0.03 |  |  | 0.15 | 0.03 | 0.12 | 0.06 |

**Table S7. Diseases associated with different sets of key EV genes based on cross-species pathway mediated gene-disease associations.** For each gene list (columns), the relevance score is the normalized relevance score computed by the third model (see Figure 1). P-values are computed by permutation tests. The top 20 most relevant diseases are reported based on the gene list of EV biogenesis and secretion.

| Disease | Extracellular vesicle biogenesis and secretion | | Extracellular vesicle biogenesis | | Exosome biogenesis | | Microvesicle biogenesis | | Exosome secretion | |
| --- | --- | --- | --- | --- | --- | --- | --- | --- | --- | --- |
|  | Relevance score | P-value | Relevance score | P-value | Relevance score | P-value | Relevance score | P-value | Relevance score | P-value |
| Transient Ischemic attack | 0.27 | 0.04 | 0.27 | 0.07 | 0.23 | 0.20 | 0.27 | 0.04 | 0.26 | 0.00 |
| Mouth neoplasms | 0.16 | 0.05 | 0.14 | 0.13 | 0.16 | 0.10 | 0.14 | 0.07 | 0.16 | 0.00 |
| Cerebral hemorrhage | 0.15 | 0.03 | 0.16 | 0.05 | 0.11 | 0.25 | 0.17 | 0.03 | 0.14 | 0.00 |
| Squamous cell carcinoma of the head and neck | 0.12 | 0.03 | 0.12 | 0.07 | 0.09 | 0.19 | 0.12 | 0.02 | 0.11 | 0.01 |
| Autoimmune lymphoproliferative syndrome | 0.11 | 0.03 | 0.10 | 0.04 | 0.05 | 0.36 | 0.11 | 0.02 | 0.12 | 0.01 |
| Proteus syndrome | 0.08 | 0.04 | 0.09 | 0.02 | 0.08 | 0.11 | 0.10 | 0.01 | 0.07 | 0.02 |
| Retinal detachment | 0.08 | 0.02 | 0.09 | 0.02 | 0.03 | 0.70 | 0.09 | 0.00 | 0.07 | 0.03 |
| Tongue neoplasms | 0.07 | 0.03 | 0.07 | 0.04 | 0.06 | 0.18 | 0.07 | 0.04 | 0.07 | 0.00 |
| Autoimmune lymphoproliferative syndrome type 2B | 0.06 | 0.01 | 0.06 | 0.03 | 0.02 | 0.43 | 0.07 | 0.01 | 0.06 | 0.02 |
| Copper-overload cirrhosis | 0.06 | 0.00 | 0.07 | 0.03 | 0.06 | 0.08 | 0.07 | 0.02 | 0.05 | 0.02 |
| Gallbladder neoplasm | 0.05 | 0.04 | 0.05 | 0.07 | 0.04 | 0.24 | 0.05 | 0.02 | 0.05 | 0.01 |
| B-cell lymphomas | 0.05 | 0.03 | 0.05 | 0.11 | 0.05 | 0.10 | 0.05 | 0.07 | 0.06 | 0.00 |
| Intermittent claudication | 0.04 | 0.03 | 0.04 | 0.03 | 0.03 | 0.15 | 0.04 | 0.03 | 0.03 | 0.01 |
| Bone diseases, developmental | 0.04 | 0.04 | 0.03 | 0.14 | 0.04 | 0.13 | 0.03 | 0.09 | 0.04 | 0.01 |
| Progressive supranuclear palsy | 0.04 | 0.04 | 0.03 | 0.10 | 0.02 | 0.27 | 0.03 | 0.11 | 0.04 | 0.02 |
| Intestinal diseases | 0.03 | 0.01 | 0.03 | 0.02 | 0.02 | 0.28 | 0.03 | 0.00 | 0.03 | 0.01 |
| Osteoarthritis, knee | 0.03 | 0.04 | 0.03 | 0.06 | 0.02 | 0.37 | 0.03 | 0.06 | 0.03 | 0.00 |
| Varicocele | 0.03 | 0.04 | 0.03 | 0.03 |  |  | 0.03 | 0.02 | 0.03 | 0.02 |
| Central neuroblastoma | 0.03 | 0.05 | 0.03 | 0.09 | 0.03 | 0.17 | 0.03 | 0.06 | 0.03 | 0.00 |
| Metaplasia | 0.03 | 0.05 | 0.03 | 0.04 | 0.02 | 0.30 | 0.03 | 0.02 | 0.03 | 0.01 |

**Table S8. Phenotypes associated with different sets of key EV genes based on cross-species pathway mediated gene-disease and disease-phenotype associations.** For each gene list (columns), the relevance score is the normalized relevance score computed by the third model (see Figure 1). P-values are computed by permutation tests. The top 20 most relevant phenotypes are reported based on the gene list of EV biogenesis and secretion.

| Phenotype | Extracellular vesicle biogenesis and secretion | | Extracellular vesicle biogenesis | | Exosome biogenesis | | Microvesicle biogenesis | | Exosome secretion | |
| --- | --- | --- | --- | --- | --- | --- | --- | --- | --- | --- |
|  | Relevance score | P-value | Relevance score | P-value | Relevance score | P-value | Relevance score | P-value | Relevance score | P-value |
| Follicular hyperplasia | 0.16 | 0.04 | 0.14 | 0.11 | 0.08 | 0.49 | 0.15 | 0.07 | 0.18 | 0.09 |
| Defective lymphocyte apoptosis | 0.16 | 0.04 | 0.14 | 0.11 | 0.08 | 0.49 | 0.15 | 0.07 | 0.18 | 0.09 |
| Chronic noninfectious lymphadenopathy | 0.15 | 0.02 | 0.13 | 0.05 |  |  | 0.14 | 0.05 | 0.16 | 0.04 |
| Increased number of peripheral CD3+ T cells | 0.15 | 0.02 | 0.13 | 0.05 |  |  | 0.14 | 0.05 | 0.16 | 0.04 |
| Increased number of CD4-/CD8- T cells expressing alpha/beta T-cell receptors | 0.15 | 0.02 | 0.13 | 0.05 |  |  | 0.14 | 0.05 | 0.16 | 0.04 |
| Increased proportion of HLA DR+ and CD57+ T cells | 0.15 | 0.02 | 0.13 | 0.05 |  |  | 0.14 | 0.05 | 0.16 | 0.04 |
| Rheumatoid factor positive | 0.15 | 0.02 | 0.13 | 0.05 |  |  | 0.14 | 0.05 | 0.16 | 0.04 |
| Increased IgG level | 0.15 | 0.02 | 0.13 | 0.05 |  |  | 0.14 | 0.05 | 0.16 | 0.04 |
| Smooth muscle antibody positivity | 0.15 | 0.02 | 0.13 | 0.05 |  |  | 0.14 | 0.05 | 0.16 | 0.04 |
| Antineutrophil antibody positivity | 0.15 | 0.02 | 0.13 | 0.05 |  |  | 0.14 | 0.05 | 0.16 | 0.04 |
| Autoimmune neutropenia | 0.15 | 0.02 | 0.13 | 0.07 |  |  | 0.14 | 0.10 | 0.16 | 0.04 |
| Platelet antibody positive | 0.14 | 0.02 | 0.12 | 0.05 |  |  | 0.13 | 0.05 | 0.15 | 0.04 |
| Increased IgA level | 0.14 | 0.03 | 0.12 | 0.09 | 0.08 | 0.34 | 0.12 | 0.13 | 0.15 | 0.05 |
| Coombs-positive hemolytic anemia | 0.13 | 0.02 | 0.12 | 0.05 |  |  | 0.13 | 0.05 | 0.14 | 0.04 |
| Defective B cell activation | 0.13 | 0.02 | 0.13 | 0.03 |  |  | 0.14 | 0.05 | 0.12 | 0.05 |
| Reduced delayed hypersensitivity | 0.13 | 0.02 | 0.12 | 0.05 |  |  | 0.12 | 0.05 | 0.14 | 0.03 |
| Increased IgM level | 0.12 | 0.03 | 0.11 | 0.05 |  |  | 0.12 | 0.11 | 0.14 | 0.04 |
| Recurrent sinopulmonary infections | 0.09 | 0.02 | 0.09 | 0.03 |  |  | 0.09 | 0.04 | 0.08 | 0.06 |
| Autoimmune hemolytic anemia | 0.09 | 0.03 | 0.08 | 0.06 |  |  | 0.08 | 0.11 | 0.09 | 0.07 |
| Decreased T cell activation | 0.07 | 0.03 | 0.07 | 0.03 |  |  | 0.07 | 0.08 | 0.06 | 0.08 |
